# CCNs and other extracellular matrix proteins: an introduction to the special issue

**DOI:** 10.1007/s12079-023-00770-x

**Published:** 2023-05-31

**Authors:** Ralf Weiskirchen

**Affiliations:** Institute of Molecular Pathobiochemistry, Experimental Gene Therapy and Clinical Chemistry (IFMPEGKC), RWTH University Hospital Aachen, Pauwelsstr. 30, Aachen, D-52074 Germany

The extracellular matrix (ECM) is a specialized, highly organized and dynamic three-dimensional network composed of a complex mixture of proteins and other molecules forming the physical scaffolding of a cell and determining the tissue architecture of organs (Rais et al., 2023). It is of fundamental importance in cell growth, cell migration, and cellular communication. It is further a reservoir for growth factors and an anchor for cell-matrix, cell adhesion, and signaling receptors (Kyriakopoulou et al. 2023). Altered composition or dysregulated ECM remodeling can result in a wide range of diseases that include tissue stiffening, connective tissue disorders, muscular dystrophy, fibrosis, and cancer. Therefore, there is hope that increasing knowledge on the mechanisms that regulate ECM composition will lead to improved diagnostics and novel strategies for repair and regeneration of affected tissues (Keane et al. 2018). In particular, the six centralized coordinating network (CCN1-CCN6) factors represent general hubs that operate through diverse signaling pathways, thereby impacting a wide array of biological properties in tissue homeostasis and malignancy (Yeger and Perbal 2021).

This Special Issue of *Journal of Cell Communication and Signaling* (JCCS) entitled “*CCNs and other extracellular matrix proteins*” contains a comprehensive editorial, 7 reviews, and 4 original research articles reporting novel concepts and major advances in our understanding of basic and clinical aspects on CCN biology. The collection of these articles demonstrates the eminent progress made in the CCN field during the last years and supports the hope that this knowledge will help establishing novel therapies for various pathologies associated with imbalance or de-regulation of CCN proteins and pathways modulated by this multifaceted protein family.

The first contribution in this Special Issue is a profound Editorial by Perbal et al. (2023) in which exciting basic principles, concepts, new views and considerations on the CCN family of protein are discussed. The article highlights important theoretical and conceptual considerations on how CCN family members can coordinate different signaling pathways. Strikingly, individual CCN members are functional “bipartite-acting” mediators, with members acting negatively and/or positively on cell proliferation and differentiation. As such, it is critical that expression of CCN members is under strict time- and tissue-specific regulation. The article further provides an extensive reference work for the CCN interactome. Importantly, the four structural modules of CCNs (i.e., insulin-like growth factor binding domain, von Willebrand factor-C domain, thrombospondin type 1 repeat domain, and carboxy-terminal cysteine knot domain) can interact with a high number of distinct ligands. Thus, it is estimated that different combinations of possible binding partners will result in nearly 9,000 liaison possibilities. Simultaneous expression of CCN members combined with the spatiotemporal availability of their putative binding partners that modulate their binding capacity to recipient cells increases the complexity in potential protein conditions to 2 × 10^22^. Finally, functional interaction of different CCNs, occurrence of biological active modules, and many other factors further increase the complexity of the CCN network. Undoubtedly, this contribution stimulates reflection and in-depth discussion and shows that individual CCNs are not lone wolves, but a pack of wolves acting together in an orchestrated, finely tuned manner, in which their interplay set the final biological opportunities of their activities.

In retinal neuronal and vascular development and function, various CCN proteins play essential function. The review by Chaqour (2023) highlights the role of the CCN-Hippo-Yes-associated protein (YAP) signaling axis in the development and stability, retinal structures, and visual function. The author discusses how alterations that prevent proper interaction of CCN1 and CCN2 with the transcriptional co-activator YAP that is central to the Hippo pathway can lead to a range of neurovascular diseases including diabetic retinopathy, retinopathy of premature, age-related macular degeneration. Consequently, the understanding how compounds of the CCN-Hippo YAP axis influence each other will provide the basis to define how these molecules can be pharmacologically or genetically manipulated in a therapeutic context.

Another example of the complexity of CCN function is described by Muromachi et al. (2023). In their original article, they showed that the bone morphogenetic protein-1 (BMP-1) induced CCN2 expression and is associated with attenuated α2,6-sialylation of several proteins in human dental pulp cells. The authors report the nuclear accumulation of β-glucosylceramidase (GBA1). This was strongly blocked by an importin-β inhibitor which further suppressed BMP-1-induced CCN2 mRNA expression. Similarly, targeted inhibition by GBA1 attenuated BMP-1-induced mRNA expression. Thus, it is most likely that some of the activities of CCN2 in chondrogenesis and osteogenesis are mediated through the BMP-1/GBA1/CCN2 axis that impacts glycosylation and activity or stability of proteins in dental pulp cells.

Li and Li (2023) systematically investigated the expression of five members of the CCN family in the developing postnatal teeth. The authors could show that the expression pattern of CCN1, CCN4, and CCN6 are quite similar, while the expression of CCN5 exhibited a unique distribution pattern. In addition, CCN3 expression was not found at all. Although the precise function of individual CCN members was not further investigated in this study, the described expression pattern suggests individual CCN members to share similar, overlapping, and specialized functions in the setting of amelogenesis, dentinogenesis, osteogenesis, and periodontal ligament homeostasis.

In the original article presented by Qin et al. (2023), the authors investigated the impact of solar-stimulated ultraviolet (UV) irradiation on the expression of CCN1 in human skin. Interestingly, the expression of CCN1 was significantly induced in skin after exposure to UV light. Laser capture microdissection indicated that CCN1 predominantly accumulated in the ECM of the dermis and not in the epidermis. Culturing dermal fibroblasts on plates enriched with high concentrations of CCN1 induced strong activation of the focal adhesion kinase (FAK) and it downstream targets paxillin and extracellular-signal regulated kinase (ERK), most likely by triggering outside-in signaling of integrin. Moreover, the expression of collagen was reduced, while the expression of matrix metalloproteinase-1 (MMP-1) was increased. Collectively, these findings suggest that UV exposure of the skin progressively promotes aging of the dermis and reduces dermal functionality.

The brief review by Xega et al. (2023) provides a concise overview of the biological activities of CCN3, CCN4, and CCN5 in regulating adiposity, liver fibrosis, and pancreatic islets. In particular, the authors highlight the fact that these CCNs play key roles in metabolic regulation. In some cases, different CCNs convey opposing functions. CCN3 and CCN4 promote for example adiposity, while CCN5 and CCN6 suppress this condition. Similarly, the family members CCN2, CCN4 and CCN5 display pro-islet effects through numerous mechanisms, while CCN3 decreases β-cell growth and insulin section. Finally, tissue fibrosis as reported in many liver disease models is largely driven by CCN2 and CCN4, while the four other family members are suggested to have anti-fibrotic effects. It might be possible that these generalizations are caused by overlapping functions of individual CCNs, but the profound phenotypes of several *ccn* gene knockout mice demonstrate that each CCN member also has specialized functions that cannot be compensated by other members.

Borkham-Kamphorst et al. (2023) analyzed the expression of CCN5 in cultures of different types of primary rat liver cells and in an experimental model of hepatic fibrosis (i.e., the bile duct ligation model). They found that CCN5 is expressed in hepatic stellate cells (HSCs), myofibroblasts, and portal myofibroblasts representing the fibrogenic cell subpopulation of the liver. In hepatocytes CCN5 expression was virtually absent. Importantly, CCN5 expression significantly increased in vitro and in vivo during hepatic fibrosis and was associated with induction of endoplasmic reticulum stress, unfolded protein response and apoptosis. Based on their findings, the authors suggest that increased CCN5 expression is an internal control mechanism counteracting overshooting fibrotic responses in pro-fibrogenic liver cells.

The review by Barkin et al. (2023) summarized the current knowledge of biological activities and molecular involvement of CCN proteins in maintenance of liver development, health, initiation and progression of hepatic diseases, and liver restoration. The discussion shows that CCNs are of fundamental importance in hepatocyte-driven liver regeneration. In particular, CCN1 and CCN2 are quickly upregulated in regenerating murine livers after partial hepatectomy. In this condition, CCN1 induces the senescence-associated secretory phenotype (SASP) in HSCs to express IL-6 and CXCL2, two crucial mediators that promote hepatocyte proliferation. CCN2 expression in hepatocytes is stimulated by Hnf4α, YAP and TGF-β and this CCN member evolves pleiotropic effect in regenerating liver tissue. In contrast, in carbon tetrachloride-induce liver damage, CCN1 expression is mainly induced in HSC and CCN2 in Hnf4α positive hepatocytes. Moreover, during hepatic fibrogenesis, CCN2 and CCN4 act pro-fibrogenic, while the other four members evolve anti-fibrotic activities. Meanwhile, CCN1-CCN4 are majorly involved in early embryogenesis, while CCN5 and CCN6 seem to be of eminent importance in hepatic differentiation. Altogether, these studies suggest that the expression of individual CCN members is fine-tuned during liver development, liver disease, and liver regeneration in parenchymal (e.g., hepatocytes) and non-parenchymal (e.g., HSC) individual liver cell subpopulations. Moreover, this contribution further demonstrates that aspects of CCN function in liver progenitor cells or oval cells during liver regeneration are still unresolved and that additional studies are needed to determine the therapeutic potential of CCN protein targeting in liver failures.

The personal perspective of Yeger (2023) provides important new ideas and concepts how CCN-based therapeutic modalities can be applied. For each CCN member, the author discusses recent findings on cancer-relevant and non-cancer-relevant issues that might be starting point for new forms of CCN-targeted therapies. Numerous strategies to suppress (knock-out studies, siRNA, shRNA, CCN-directed antibodies, translational downregulation, miRNAs, CCN-targeted peptides, CCN-mediated nanotechnology) or overexpress (transcriptional stimulation, tea extracts or compounds, encapsulated CCNs, transient CCN or CCN module gene transfer, CCN-loaded exosomes) CCN expression or activity are established. Some of them were already successfully used to interfere with reactive oxygen species formation, wound healing, matrix remodeling, cellular senescence, tissue aging, cell adhesion, migration, proliferation, differentiation, survival, epithelial-mesenchymal transition (EMT), and composition of the tumor microenvironment and immune evasion. Since the biological alterations associated with imbalanced CCN protein expression are manifold, it is obvious that each strategy to silence or activate CCN functionality has potential caveats that must be addressed. Encouragingly, a humanized anti-CCN2 antibody is currently undergoing phase III clinical trials and individual CCNs or modules thereof have already received diagnostic value in certain diseases.

In focus of the review by Kubota et al. (2023) is CCN3 that plays important role in the development, growing, and aging of cartilage. The authors emphasize the Yin/Yang concept in the foreground of their discussion and provide impressive examples of opposing activities of CCN3 and CCN2 in both physiological and pathological processes. There are several examples in which CCN2 acts as a physiological brake that dims down the expression of CCN3. Exemplarily, chondrocytes isolated from the rib cages of mice lacking CCN2 show elevated expression of CCN3 accompanied with impaired glycolysis and drastically reduced cellular ATP quantities. In this setting, the induction of CCN3 through impaired glycolysis is most likely mediated by the regulatory factor binding to the X-box (RFX1) that stimulates CCN3 expression by binding to a proximal proximal *CCN3* promoter region. Elevated CCN3 then reduce cell proliferation and assist cellular survival by reducing energy expenditure, while maintaining the quiescence and stemness of chrondrocytes. Consequently, CCN3 is a kind of biological guard that prevents “overwork” by chondrocytes, while CCN2 stimulates chondrocyte proliferation in articular, auricular and growth-plate cartilage.

Modified expression of CCNs can also be induced by physical shear stress. In general, the cell microenvironment is formed by physical (shear stress), biochemical (cell interactions), and physicochemical (temperature, oxygen saturation, pH, carbon dioxide concentration). In their review, Wang et al. (2023) comprehensively discuss the impact of shear stress on CCN regulation with special emphasis on the cardiovascular and skeletal systems. In endothelial cells, CCN1 and CCN2 are induced by oscillatory shear stress, while the expression of both CCNs is suppressed by laminar shear stress. Likewise, the expression of CCN3 is induced in cultured ECs by laminar shear stress. Therefore, it is not surprising that arterial disease, dysfunctional endothelium, and neointimal hyperplasia are associated with modified expression of individual CCNs. Similarly, mechanical stimulation, mechanical load, and remodeling of the bone are associated with significant alterations of CCN expression. Of course, the underlying mechanisms and pathways are not fully understood yet, but it is obvious that a deeper knowledge of the overall processes might have significant clinical implications.

Finally, the review by Monsen and Attramadal (2023) critically discusses structure-function relationships of CCNs. In particular, comprehensive insights are presented showing that CCNs are more than just simple scaffold protein hubs that provide interaction interfaces for other proteins. The authors discuss that CCNs are signaling proteins on their own, which have important functions in autocrine or paracrine signaling. Their activity is controlled by the microenvironment of the local ECM, proteolytic activation, and interaction with numerous receptors and co-receptors. Structural insights obtained by crystal structure and artificial intelligence fold predictions have provided deep information about the three-dimensional structure of individual CCN modules. Interestingly, ample evidence is now available showing that individual CCN domains formed by endopeptic cleavage can have strong activating or inhibitory activities. In particular, the C-terminal fragments of CCN1, CCN3, and CCN5 are fully active and can recapitulate previously reported functions of their full length counterparts. It will now be interesting to see how the knowledge of structure-function predictions will help fostering the search for CCN-based therapeutics.

To sum up, this Special Issue provides a unique compilation of contemporary findings and new concepts on CCN biology from leading laboratories originating from 9 countries and working in this research area. Although the individual contributions demonstrate the relentless progress in field of this versatile protein family, there is still a gap in translating basic laboratory findings into human applications and potential treatments. However, there is hope that the new findings, ideas and concepts presented in this Special Issue will help to foster the process of clinical translation into therapies and development of new diagnostic tests.
